# Effect of CAD/CAM Ceramic Thickness on Shade Masking Ability of Discolored Teeth: In Vitro Study

**DOI:** 10.3390/ijerph182413359

**Published:** 2021-12-18

**Authors:** Passent Ellakany, Marwa Madi, Nourhan M. Aly, Zainb S. Al-Aql, Maher AlGhamdi, Abdulrahman AlJeraisy, Adel S. Alagl

**Affiliations:** 1 Department of Substitutive Dental Sciences, College of Dentistry, Imam Abdulrahman Bin Faisal University, Dammam 32210, Saudi Arabia; 2 Department of Preventive Dental Sciences, College of Dentistry, Imam Abdulrahman Bin Faisal University, Dammam 32210, Saudi Arabia; mimadi@iau.edu.sa (M.M.); aalagl@iau.edu.sa (A.S.A.); 3 Department of Pediatric Dentistry and Dental Public Health, Faculty of Dentistry, Alexandria University, Alexandria 21527, Egypt; nourhanovic@gmail.com; 4 Dental Sciences, King Abdulaziz Medical City, National Guard Health Affairs, Jeddah 22230, Saudi Arabia; aqlz@ngha.med.sa; 5 College of Dentistry, Imam Abdulrahman Bin Faisal University, Dammam 32210, Saudi Arabia; 2170003681@iau.edu.sa (M.A.); 2170002764@iau.edu.sa (A.A.)

**Keywords:** lithium disilicate, leucite reinforced, shade, esthetics, translucency, CAD/CAM

## Abstract

Shade matching is a common challenge that dentists face during fabrication of esthetic dental restoration. Thus, the aim of the current study was to assess the masking ability of two types of CAD/CAM ceramics for gaining high esthetic prosthesis. This in vitro study used a total sample size of 66 lithium disilicate (LD) and leucite reinforced (LR) CAD/CAM ceramics sub-grouped into three thicknesses: 0.5, 1, and 1.5 mm. Nine shades of natural dentin die materials were prepared as a replica of the underlying tooth structure. The difference in color (ΔE) and translucency parameter (TP) were assessed for both tested ceramics at the three thicknesses. One-way ANOVA was performed to compare the three thicknesses of each ceramic, followed by multiple pairwise comparisons between both ceramics. LR had significantly higher ΔE than LD at all thicknesses used unlike the case in TP. Thickness of 0.5 mm exhibited the highest ΔE and TP, while 1.5 mm thickness showed the lowest ΔE and TP in both ceramics. Increase in ceramic thickness had a great impact on both color masking ability of the underlying tooth structure and its translucency. The higher the ceramic thickness, the better the masking ability and the lower the translucency was reported.

## 1. Introduction

Fabrication of dental restorations requires fulfillment of functional needs such as mastication, occlusion, speech, and esthetics [1]. Patients are currently greatly concerned with esthetics more than any other factor. This makes fabrication of dental restorations challenging for both dentists and lab technicians to achieve the optimum outcome that satisfies patients’ needs [2].

Dental ceramics are among the most esthetic restorative materials used in dentistry [1,3]. They are characterized by having high compressive strength, resistance to cracking, crack propagation stability, biocompatibility, and optical properties that match natural teeth features [4,5]. The optical characteristics of dental ceramics are affected by several factors; the type and translucency of ceramics, the shade of the underlying tooth structure, and the luting cement [6,7,8]. Recently, digital dentistry has widely spread allowing fabrication of CAD/CAM ceramic restorations that have superior properties such as high translucency and esthetics. This helps in minimizing chairside time, facilitating shade selection and communication between dentist and lab technician, and reducing the fabrication defects produced from conventional fabrication methods such as voids, cracks, and firing problems [9,10,11].

Additionally, digital shade selection can minimize the drawbacks of subjective shade selection that can be affected by a dentist’s own vision, color blindness, surrounding environment (including colors of walls and ceiling and type of light available in the dental clinic), patient’s skin color, age, gender, and the duration consumed in shade selection where extended duration might lead to eye fatigue [12,13,14].

Recent types of dental ceramics enable clinicians to fabricate monolithic esthetic restorations with a conservative tooth preparation achieving the required shade [9]. However, translucency of the restoration depends on the amount of light transmission through the restoration, which is affected by the shade of underlying tooth structure, cement, and thickness of the ceramic used, and its composition [15,16]. Lithium disilicate (LD) and leucite-reinforced (LR) glass ceramics are characterized by high translucency. They are used in fabrication of esthetic monolithic restorations due to the reduced refractive index despite their excessive crystalline composition [11,17]. However, LD is more recommended than LR ceramics as they have better mechanical and optical properties with lower failure rates [11,18].

Limited studies have assessed the clinical influence of dental ceramic thicknesses and composition on the masking ability and translucency of definitive prosthetic restorations [19,20]. Moreover, maintaining a proper balance between translucency and ceramic thickness is crucial especially in cases of dark discoloration of the underlying tooth structure or amalgam restorations [21]. Furthermore, thin ceramic restorations as dental veneers are favorable for several patients that seek high esthetic smile demand; however, their color properties are still sparse [22,23].

The aim of the current study was to evaluate the ability of different ceramic thicknesses of LD and LR CAD/CAM ceramics on masking the underlying tooth structure to achieve optimum esthetics and translucency. The null hypothesis of the current study states that different ceramic thickness and composition do not have a great impact on shade masking ability of underlying discolored teeth.

## 2. Materials and Methods

### 2.1. Samples Preparation

**Figure 1:**
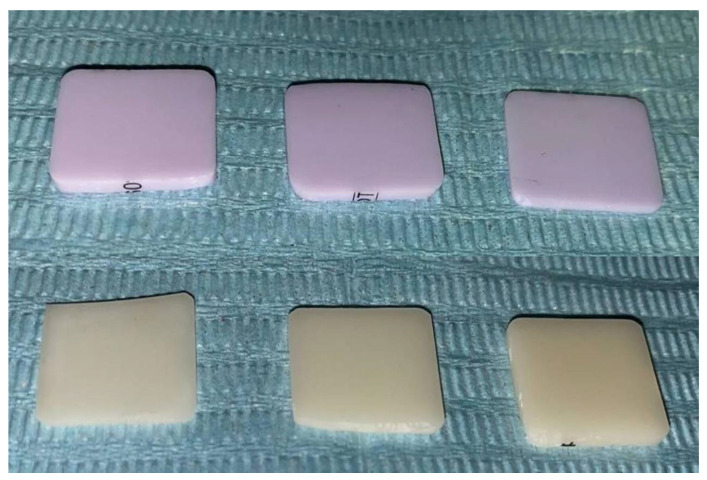
LD and LR CAD/CAM ceramic specimens of 0.5, 1, and 1.5 mm thicknesses.

The current in vitro study evaluated the effect of different thicknesses of lithium disilicate and leucite-reinforced CAD/CAM ceramics on the masking ability of underlying discolored teeth. Ceramics used in this study were LD (IPS Emax CAD, Ivoclar Vivadent, Schaan, Liechtenstein) and LR (IPS Empress CAD, Ivoclar Vivadent, Schaan, Liechtenstein) shade A1 low translucency CAD/CAM ceramic blocks. Both types of ceramic blocks were sectioned into eleven samples of three different thicknesses (0.5 mm, 1 mm, and 1.5 mm thick) using a precision sectioning machine (Isomet 1000, Buehler; Lake Bluff, IL, USA) with a total sample size of 66 (Figure 1).

Polishing of the ceramic specimens was performed using medium grit of silicon carbide discs on a polishing machine (MetaServ 250 Grinder-Polisher with Vector Power Head, Buehler, IL, USA) under wet circumstances. The final thickness of the specimens was measured by a digital caliper (Mitutoyo Corp, Kawasaki, Japan) to be within 0.05 mm thick. Glazing of the samples was performed by applying a thin glazing layer on the surface of one side of every specimen, followed by firing at 403 °C for 1 min then subjected to drying for 6 min. The temperature was increased to reach 770 °C, and then the specimens were placed under vacuum for 90 s. All specimens were washed by distilled water, left to dry then stored in a sealed box at room temperature for measuring color values [19].

### 2.2. Underlying Background Formation

**Figure 2:**
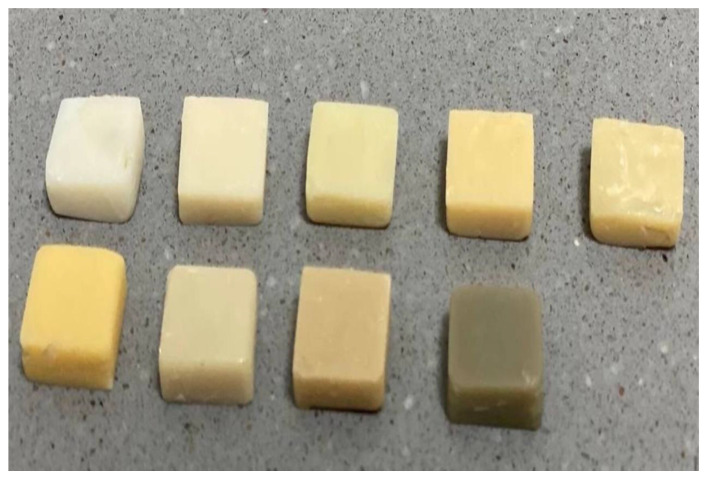
Natural dentine die specimens of 9 different shades.

Nine different shades of natural dentin die materials were prepared in a putty index of 5 mm thickness by incremental condensation of dentin in the form of discs, and polymerization was performed using a light emitting diode (LED) curing unit (Bluephase, soft start mode, Ivoclar Vivadent, Schaan, Liechtenstein) at an intensity of 1200 mw/cm^2^ (Figure 2).

### 2.3. Color Measurement of Different Ceramic Thicknesses

**Figure 3:**
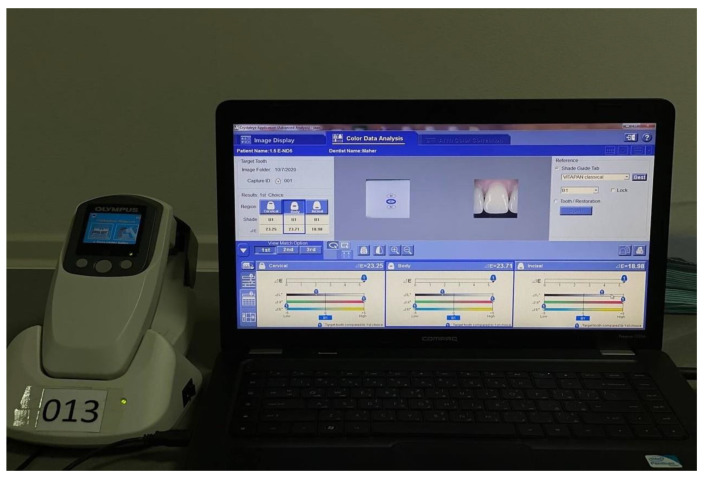
Shade detection of ceramic specimens using Crystaleye Olympus spectrophotometer.

The shade of different thicknesses of both ceramics against the nine shades of natural dentine die were measured by the spectrophotometer (SpectroShade, MICRO, Serial N HDL1407, MHT, Arbizzano di Negrar, Verona, Italy; Crystaleye, Olympus, Tokyo, Japan) (Figure 3). Then, each thickness from both types of ceramics used was measured against a white and black background to serve as a positive and negative control using the spectrophotometer. Thereafter, the nine natural dentin dies were placed behind the ceramic specimens of the three thicknesses and color was measured by the same device. A total of 45 color measurements (*n* = 5 color measurements × 9 dentine shades = 45 readings) were recorded for each 0.5 mm, 1 mm, and 1.5 mm thickness of the LD and LR CAD/CAM ceramics. The color coordinates were recorded on the middle of the specimen three times in the form of L, a, and b values and then ΔE value was calculated. The tenth and eleventh ceramic specimens of each thickness of LD and LR ceramics were used in independently recording five translucency readings against white and black backgrounds.

Measurement of color was performed through the use of the CIELAB (L*a*b) [24] color model in the form of color difference where the “L” value refers to lightness or darkness (zero means completely black, 100 means completely white), the “a” value refers to red/green chroma (positive “a” referred to redness, negative “a” referred to greenness), and the “b” value denotes yellow/blue chroma (positive “b” referred to yellowness, negative “b” referred to blueness).

The color difference between the actual color of the ceramic specimen and the specimen after adding a different dentin background was measured using the following equation [25,26]:

ΔE∗ = [(ΔL∗)^2^ + (Δa∗)^2^ + (Δb∗)^2^]^1/2^ (1)

Difference in color (ΔE) recorded <2.6 is considered visually undetectable (ideal shade match). However, if the color difference was found to be more than 3.3, this means that the shade detected is not matching or clinically unacceptable [25].

### 2.4. Measurement of Translucency Characteristic 

The translucency of ceramic specimens was evaluated using translucency parameter (TP) equation. Calibration of spectrophotometer was achieved by applying white. Color coordinates of each sample were independently measured on white and black backgrounds. The color coordinates were measured in the form of CIELAB parameters (L*, a*, and b*) under the same light source used throughout the study [3,27,28].

TP = [(LW∗–LB∗)^2^ + (aW∗–aB∗)^2^ + (bW∗–bB∗)^2^]^1/2^ (2)

where TP: translucency parameter; W: white background; B: black background.

### 2.5. Statistical Analysis

Normality was checked for all variables using descriptive statistics, plots, and normality tests. All variables showed normal distribution, so means and standard deviations (SD) were calculated, and parametric tests were used. Comparison between the two study groups was conducted using an independent samples t-test, while comparison between the different thicknesses of each material was conducted using one-way ANOVA, followed by multiple pairwise comparisons using Bonferroni-adjusted significance level. Two-way ANOVA was performed to determine the influence of material and thickness on color change (ΔE) and translucency parameter (TP). Significance was set at *p* value < 0.05. Data were analyzed using IBM SPSS for Windows (Version 23.0) (IBM, Armonk, NY, USA).

## 3. Results

Table 1 shows that LR had significantly higher ΔE than LD of different thicknesses and against all nine dentin shades (*p* < 0.05), with the lowest ΔE values against dentin shade 1 and the highest values against dentin shade 9. The lowest mean ± SD ΔE was reported in 1.5 mm thickness LD against natural dentin shade 1 (1.56 ± 0.08), while the highest ΔE was for 0.5 mm thickness LR against natural dentin shade 9 (16.96 ± 0.02).

Table 2 highlights the significant difference in ΔE between LD and LR of different thicknesses (*p* < 0.05) with higher mean scores for LR material. There was a statistically significant difference between ΔE of different thicknesses of the same material (*p* < 0.001) with the highest ΔE reported in 0.5 mm and the lowest ΔE reported in 1.5 mm for both materials.

**Figure 4:**
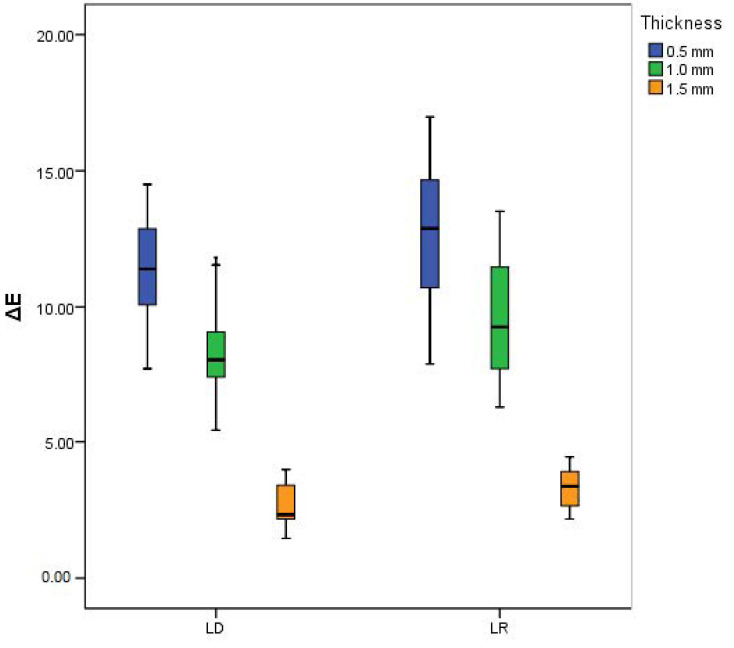
Color difference (ΔE) of LD and LR at different thicknesses.

Table 3 and Figure 4 represent the influence of both materials and thicknesses on ΔE adjusted for the differences in natural dentin shade. LR had significantly higher ΔE than LD (adjusted mean = 8.54 and 7.42, for LR and LD, respectively, *p* < 0.001). There was a statistically significant difference between ΔE of different thicknesses (adjusted means = 12.00, 8.97, and 2.98 for 0.5 mm, 1 mm, and 1.5 mm, respectively, *p* < 0.001).

The middle line of the boxplot represents the median (50th percentile), the box represents the interquartile range (25th and 75th percentiles), and the whiskers represent minimum and maximum values (see Figure 4).

**Figure 5:**
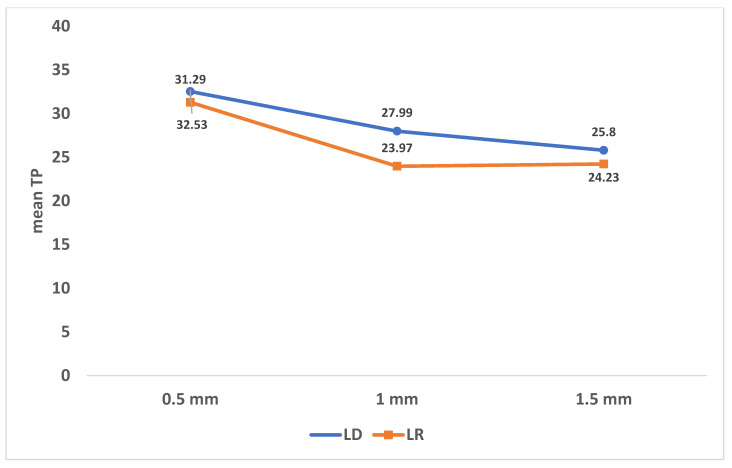
Translucency parameter (TP) of LD and LR at different thicknesses.

Table 4 and Figure 5 show that LD had statistically higher TP than LR at different thicknesses (*p* < 0.05). There was a statistically significant difference between TP of different thicknesses of the same material (*p* < 0.001), with the highest mean TP reported in 0.5 mm and the lowest in 1.5 mm thickness.

Table 5 represents the influence of both materials and thicknesses on TP adjusted for the differences in natural dentin shades. LD had significantly higher TP than LR (adjusted mean of LD = 29.91 and of LR = 28.36, *p* = 0.003). There was a statistically significant difference between TP of different thicknesses (adjusted means = 31.91, 25.98, and 25.02 for 0.5 mm, 1 mm, and 1.5 mm, respectively, *p* < 0.001). Post hoc pairwise comparisons showed a non-significant difference in TP between 1 mm and 1.5 mm thicknesses.

## 4. Discussion

Color and translucency parameters strongly influence the esthetic outcome of dental restorations [29]. Synchronization of ceramic shade with natural teeth shade is even more challenging because of the underlying background color. The present study evaluated the masking ability of three different thicknesses of LD and LR CAD/CAM ceramics against nine different dentin backgrounds to simulate different clinical situations. A wide range of different shades of underlying substrates were used in the current study to provide greater validation on the masking ability and translucency of dental ceramics, unlike the use of a single shade of composite resin as substrate. Findings show that LD specimens of all thicknesses exhibited higher masking ability than LR, thus, the study hypothesis was rejected.

Sethakamnerd et al. [30] showed that the color of the substrate affects the outcome of the definitive color. This explains using nine different dentin shades as backgrounds in the current study in order to mimic natural dentin color and evaluate their effect on the final color of the prosthetic restoration.

Not all ceramic types and thicknesses effectively masked the black background. These results agree with the findings of Shono and Nahedh [31], who showed that no ceramic could entirely conceal a black background. However, color-masking ability was enhanced when thickness was increased from 1.0 to 1.5 mm, which is in agreement with our findings [31].

The variation abilities among individuals in recognizing color differences was reported in the literature as follows: ΔE less than 1 is undetectable by the human eye, ΔE more than 1, but less than 3.3 is detectable by experts and clinically acceptable, and ΔE more than 3.3 is detectable by patients and unskilled observers and is considered clinically unacceptable [32]. The current results show a significant difference between ΔE of different thicknesses of the same material (*p* < 0.001) with the highest mean value for 0.5 mm, but decreasing ceramic thickness resulted in higher ΔE values. Mean ΔE for both materials were <3.3 at 1.5 mm thickness (2.62 and 3.27). This means that both evaluated ceramic samples show acceptable values for masking a simulated tooth substrate at 1.5 mm thickness. However, thinner samples show higher ΔE (LD: 11.36, 8.21 and LR: 12.58, 9.51 for 0.5 and 1 mm thicknesses, respectively), indicating poor masking ability. These findings were in agreement with previous studies where ΔE values were significantly influenced by both the ceramic material and sample thicknesses [31,33].

According to the manufacturer’s instructions, it is suggested that at least 2 mm ceramic thickness of LD should be used to mask the effect of the underlying discolored tooth or abutment [32]. However, the current findings show that LD CAD/CAM ceramics of 1.5 mm thickness could provide efficient masking for different shades of the underlying substrate. This finding is important, because in many clinical cases, it is difficult to achieve a 2 mm axial reduction without compromising the strength of the remaining tooth structure. It should be kept in mind that the possibility of achieving maximum esthetics with conservative tooth preparation is a critical factor [34].

Many studies identified TP as the primary factor in controlling aesthetics that affects the selection of veneer materials [16,28,34,35,36,37,38]. Crystals in ceramic matrices tend to control the relative amount of light absorbed, reflected, and transmitted [39]. The higher the number of crystals in a glassy matrix, the lower the reported translucency of the ceramic. In order to obtain a natural-looking esthetic restoration, the TP values of veneer materials should be close to that of natural enamel [40]. The current results show that TP of LD was significantly higher than that of LR. When thickness increased from 0.5 to 1.5 mm, TP decreased for both ceramics as reported in other studies [29,41,42]. The difference in TP between LD and LR could be due to the needle-like crystal structure of LD glass–ceramics, which offer excellent optical properties in addition to the presence of zircon-based pigments that enhance optical properties of ceramics [43]. Moreover, using CAD/CAM ceramic ingots with low translucency was based on previous findings reporting that low translucency LD exhibited better masking ability than high translucency ceramics. The current findings confirm that low translucency ceramic ingots can be chosen to provide better shade matching to natural dentition in an easier and less fabricated time than multilayered ceramics as reported in Pande and Kolarkar [44].

Significant differences were also observed between the TPs of different thicknesses used for both LD and LR. These results agree with previous studies [17,19,41,45,46], which reported that masking ability and shade reproduction of ceramics might be affected by several factors such as thickness, composition, and translucency [47], where LD ceramics represented better translucency when compared to other ceramics [48]. Moreover, Wang et al. [49] measured TPs of zirconia and LD glass-based ceramics at different thicknesses, indicating that both materials and their thicknesses affected the translucency of the ceramic restorations.

The strengths of the current study were the use of a digital shade guide (digital spectrophotometer) by a single trained investigator to provide valid shade readings and exclude any visual variations in shade selections between researchers or within the same researcher. In addition, was the use of nine different natural dentin shades to mimic the various discolorations that could be encountered among natural dentition of various individuals. However, the study had some limitations including carrying out the study in dry in vitro conditions, which do not resemble the oral cavity circumstances of temperature or moisture. Using flattened specimens rather than tooth-shaped veneers might also decrease relevance to the actual clinical situation.

## 5. Conclusions

LD ceramics exhibited better masking ability and translucency than LR ceramics. The lower the ceramic thickness, the better translucency and less masking ability was shown. Using low translucency monolithic CAD/CAM ceramic blocks might also provide proper esthetic restoration with minimum conservative natural tooth preparation and optimum masking ability.

## Figures and Tables

**Table 1:** Comparison of color change (ΔE) between LD and LR of different thicknesses against nine natural dentin shades.

Thickness	Natural Dentin Shade	LD	LR	*p* Value
		Mean ± SD		
0.5 mm	N1	7.80 ± 0.05	7.93 ± 0.05	0.003 *
	N2	10.06 ± 0.008	9.60 ± 0.07	<0.001 *
	N3	10.09 ± 0.009	10.74 ± 0.07	<0.001 *
	N4	10.93 ± 0.07	11.59 ± 0.07	<0.001 *
	N5	11.37 ± 0.08	12.90 ± 0.06	<0.001 *
	N6	11.53 ± 0.07	13.29 ± 0.03	<0.001 *
	N7	12.84 ± 0.06	14.62 ± 0.07	<0.001 *
	N8	13.41 ± 0.08	15.88 ± 0.03	<0.001 *
	N9	14.43 ± 0.06	16.96 ± 0.02	<0.001 *
1 mm	N1	5.56 ± 0.09	6.34 ± 0.05	<0.001 *
	N2	5.84 ± 0.07	7.05 ± 0.01	<0.001 *
	N3	7.42 ± 0.06	7.72 ± 0.08	<0.001 *
	N4	7.91 ± 0.06	8.46 ± 0.06	<0.001 *
	N5	8.00 ± 0.002	9.24 ± 0.07	<0.001 *
	N6	8.83 ± 0.06	10.75 ± 0.06	<0.001 *
	N7	9.07 ± 0.009	11.41 ± 0.07	<0.001 *
	N8	9.92 ± 0.08	12.85 ± 0.06	<0.001 *
	N9	11.62 ± 0.09	13.48 ± 0.03	<0.001 *
1.5 mm	N1	1.56 ± 0.08	2.20 ± 0.02	<0.001 *
	N2	1.85 ± 0.07	2.29 ±0.03	<0.001 *
	N3	2.17 ± 0.03	2.65 ± 0.07	<0.001 *
	N4	2.21 ± 0.02	2.93 ± 0.08	<0.001 *
	N5	2.30 ± 0.08	3.36 ± 0.05	<0.001 *
	N6	2.88 ± 0.03	3.79 ± 0.04	<0.001 *
	N7	3.36 ± 0.07	3.89 ± 0.05	<0.001 *
	N8	3.62 ± 0.04	4.24 ± 0.07	<0.001 *
	N9	3.91 ± 0.07	4.40 ± 0.05	<0.001 *

* Statistically significant at *p* value < 0.05 *.

**Table 2:** Comparison of color change (ΔE) between LD and LR of different thicknesses.

Thickness	LD	LR	T-Test *p* Value
	Mean ± SD		
0.5	11.38 ± 1.91 a	12.61 ± 2.81 a	0.02 *
1	8.24 ± 1.82 b	9.70 ± 2.44 b	0.002 *
1.5	2.65 ± 0.79 c	3.30 ± 0.79 c	<0.001*
Average	7.42 ± 3.95	8.54 ± 4.47	0.03 *
One-way ANOVA *p* value	<0.001 *	<0.001 *	

* Statistically significant at *p* value < 0.05 *; a, b, and c: different letters denote statistically significant differences between different thicknesses (0.5 mm vs. 1 mm vs. 1.5 mm) within each group using Bonferroni-adjusted significance levels.

**Table 3:** Two-way ANOVA for association of ceramic material and on color change (ΔE).

		Adjusted Mean (SE)	95% CI	*p* Value
Material	LD	7.42 (0.07)	7.28, 7.57	<0.001 *
	LR	8.54 (0.07)	8.40, 8.68	
Thickness	0.5 mm	12.00 (0.09) a	11.82, 12.17	<0.001 *
	1 mm	8.97 (0.09) b	8.80, 9.15	
	1.5 mm	2.98 (0.09) c	2.80, 3.15	

Scheme 0. Model F: 608.51, *p* value < 0.001 *, adjusted R2: 0.96. Model was adjusted for natural dentin shade differences; a, b, and c: different letters denote statistically significant differences between different thicknesses (0.5 mm vs. 1 mm vs. 1.5 mm) using Bonferroni-adjusted significance levels.

**Table 4:** Comparison of translucency parameter (TP) between the two study groups at different thicknesses.

Thickness	LD	LR	T-test *p* Value
	Mean ± SD		
0.5	32.53 ± 0.51 a	31.29 ± 0.07 a	0.005 *
1	27.99 ± 0.14 b	23.97 ± 0.13 b	<0.001 *
1.5	25.80 ± 0.07 c	24.23 ± 0.55 c	<0.001 *
Average	28.36 ± 2.35	26.91 ± 4.13	0.25
One-way ANOVA *p* value	<0.001 *	<0.001 *	

* Statistically significant at *p* value <0.05; a, b, and c: different letters denote statistically significant differences between different thicknesses (0.5 mm vs. 1 mm vs. 1.5 mm) within each group using Bonferroni-adjusted significance levels.

**Table 5:** Two-way ANOVA for association of ceramic material and thickness and TP.

		Adjusted Mean (SE)	95% CI	*p* Value
Material	LD	29.91 (0.31)	26.28, 27.55	0.003 *
	LR	28.36 (0.31)	27.73,29.00	
Thickness	0.5 mm	31.91 (0.39) a	31.14, 32.69	<0.001 *
	1 mm	25.98 (0.39) b	25.21, 26.76	
	1.5 mm	25.02 (0.39) b	24.24, 25.79	

Model F: 68.79, *p* value < 0.001 *, adjusted R^2^: 0.87. * Statistically significant at *p* value < 0.05; a and b: different letters denote statistically significant differences between different thicknesses using Bonferroni-adjusted significance levels.

## Data Availability

Data is available upon request from the corresponding author.
